# Rapid Visual Tests: Fast and Reliable Detection of Ochratoxin A

**DOI:** 10.3390/toxins2092230

**Published:** 2010-08-26

**Authors:** Ingrid Bazin, Elodie Nabais, Miguel Lopez-Ferber

**Affiliations:** Ecole des Mines d’Ales, 6 av. de Clavieres 30319, Ales cedex, France; Email: elodie.nabais@mines-ales.fr (E.N.); Miguel.Lopez-Ferber@mines-ales.fr (M.L.-F.)

**Keywords:** ochratoxin A, colorimetric test, lateral flow test, flow-through test, clean-up immunoassay

## Abstract

This paper reviews the early detection strategies that have been employed for the rapid monitoring of ochratoxin A (OTA) contamination of food. OTA, a mycotoxin mainly produced by some *Aspergillus* and *Penicillium* species, is found in cereals, coffee, wine, pork and grapes. To minimize the entry of this mycotoxin into the food chain, rapid diagnostic tools are required. To this end, the potential use of lateral flow devices has also been developed. In this study, we analyze the robustness of test strips using published methods for colorimetric detection. Different test formats are discussed, and challenges in the development of lateral flow devices for on-site determination of OTA, with requirements such as robustness, speed, and cost-effectiveness, are discussed.

## 1. Introduction

Rapid diagnostic assays were originally developed in the medical and clinical sectors; the urine glucose and pregnancy test strips were the first to be commercialized. The success of these tests, due to their speed and ease of use, led to their implementation in other fields, such as in the animal health and foodstuff industries. Since then, many laboratories have developed rapid analysis systems for detecting pathogens, allergens, drug residues and mycotoxins.

Ochratoxin A (OTA) is one of the mycotoxins (a natural toxic secondary metabolite) produced by several species of the fungi *Penicillium* and *Aspergillus*. This toxin represents a risk for human and animal health when ingested through contaminated food. It is cytotoxic, carcinogenic, mutagenic and immunosuppressive [1,2]. Thus, regulations are strengthening for ochratoxin A in foodstuffs (EC 105/2010 amending regulation EC 1881/2006 and EC 2006/576/EC, European Commission Regulation [3,4]) and tests for mycotoxins are exhibiting increased success (Table 1).

**Table 1:** Maximum accepted levels of Ochratoxin A in various foods in Europe (EC 105/2010).

Products	Max. Accepted Level (µg/kg)
Crude cereals	5
Processed cereals	3
Dried raisin	10
Roasted coffee	5
Soluble instant coffee	10
Wine (red, white, rose) and raisin derived products	2
Raisin juice and derived products	2
Grape must	2
Baby foods and cereal based baby foods	0.5
Probiotics	0.5
Liquorice root	20
Liquorice extract	80
Spices	30 *

* Maximum accepted level is 30 from 1 July 2010–30 June 2012; then 15 as of 1 July 2012.

Validated standard methods for the detection of ochratoxin A are based on chromatographic techniques with fluorescence detection due to the fact that OTA possesses natural fluorescence [5,6]. However, these methods are expensive and time-consuming so there is a need for simplified procedures. Rapid screening tests such as biosensors [7,8,9] and enzyme-linked immunosorbent assays (ELISA)[10] are emerging. A further development is their simplification, such as colored immuno-tests, like rapid disposable membrane-based assay tests or clean-up tandem immune assay column. These tools tend to be portable, low cost, and easy to use, giving results that can be interpreted by non-specialists. 

Rapid disposable membrane-based assays have been developed in multiple formats like dip sticks tests, strip tests and flow-though tests. Dip stick tests are simplified ELISA based tests, requiring 30 minutes to three hours. For strip tests and flow-though test systems, the total time required is 5–10 minutes [11].

This review will focus on membrane-based strip tests, flow-through tests and another system that combines solid phase clean-up and immunoassay in one device.

## 2. Biosensors

As mycotoxin contamination usually occurs in trace amounts ranging from nanograms to micrograms per gram of foodstuff, sensitive and accurate analytical methods for OTA determination are highly desirable. Other analytical methods, such as capillary electrophoresis [12], radioimmunoassay [13] and enzyme-linked immunosorbent assay [14], have been developed. More recently, several immunosensors have emerged for OTA detection including optical waveguide light mode spectroscopy (OWLS)[15], fluorescent biosensor arrays [16,17], and electrochemical immunosensors [9,18]. The development of OTA electrochemical immunosensors is based on different OTA immobilization procedures. OTA electrochemical immunosensors can be based on screen-printed gold electrodes modified with a layer of 4-nitrophenyl diazonium salt. This technique was recently reported and a detection limit of 12 ng/mL was achieved [19]. Another label-free electrochemical immunosensor that was developed on modified gold electrodes for sensitive detection of OTA was also recently published [20]. The direct electrochemical oxidation of OTA is also possible using voltammetry at a vitreous carbon electrode [21,22]. A surface plasmon resonance based sensor for the detection of OTA has also been described [7]. The use of molecular imprinted polymer (MIP) comprising cavities that can be considered as antibody mimics have been developed for the analysis of OTA from cereal extracts [23]. However, these methods are expensive, need specific instruments and cannot be used by a non-scientific technician.

## 3. Membrane-Based Test Strip

The test strip, also called a lateral flow device or immunochromatographic strip (ICS) test, is based on a membrane which contains immobilized antibodies. The test strip has been popular for diagnostic tests since its introduction in the late 1980s. 

**Figure 1:**
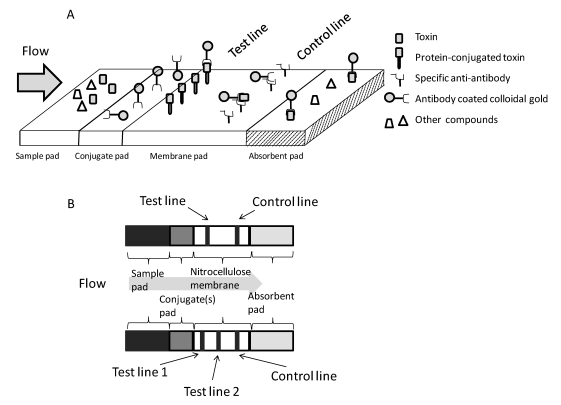
Competitive assay in test strip format (free-standing test strip). (**A**) The principle of the method, modified from [11]. The toxin recognition sites of the specific antibody conjugated to a detection system (usually colloidal gold) located in the conjugated pad will be blocked by the toxin present in the sample, thus preventing the antibody from being fixed on the test line. In the control line, an anti-antibody (usually goat anti-mouse antibody) will retain charged and uncharged conjugated antibodies, thus providing the positive control. The intensity of the test line will be inversely correlated to the toxin concentration in the sample. (**B**) Schematic diagram of simple and multiple detection strips. The two test lines contain different toxins conjugated to the membrane-bound protein. The conjugate pad contains specific antibodies to each of the corresponding toxins.

Lateral flow tests are used for the specific qualitative or semi-quantitative detection of many substances including antigens, antibodies, and even mycotoxins. The development of rapid test systems is critical for the control and the determination of contaminants such as mycotoxins in food prior to or during production. However, major restrictions arise from the matrix sample (one of the most difficult is red wine), which negatively affects both the selectivity and the sensitivity of the test [24]. One or several mycotoxins can be tested on the same strip simultaneously [25,26]. For OTA detection, the test strip is a competitive assay, as shown in Figure 1A. After 10 to 15 minutes, one or two lines become visible: one for a positive result and two for a negative result. One line, the control line, will therefore always be visible regardless of the presence of the targeted analyte, to confirm the correct development of the test. The test plate is composed of three parts, namely the sample pad, conjugate pad and reaction membrane, as shown in Figure 1A and B.

### Lateral-Flow: How Does It Work?

The test strip is a one step procedure. The liquid sample to be analyzed is placed onto the sample pad. The membrane pads are usually nitrocellulose based [25,26,27,28]. The reagent membrane contains the immobilized specific antibodies and the labeled antibodies. With the addition of the sample, the reacting molecules are solubilized. When solubilized, they combine with OTA in the sample. Then, capillary action draws the fluid mixture towards the reaction membrane. A variety of reagents can be used for visualizing the antigen/antibody interaction; colloidal gold is most often used in test strips developed for OTA [25,26].

These test strips are semi-quantitative with different visual limits of detection (LOD) in function of the nature of the sample. In the first developed test, the detection limit was 500 ng/mL OTA [28]; nowadays, the cut-off level has dropped to 1 ng/mL (Table 2). Indeed, these immunostrips provided a cut-off response for the OTA level adjusted to the most stringent limit that the European Community has fixed for foodstuffs (Table 1 and Table 2).

**Table 2:** Visual detection limit of different test strips developed for Ochratoxin A.

Visual Detection Limit (ng/mL)	Total Assay Time (min)	Sample	References
2.5	15	Maize	[26]
10	10	Cereal and soybean	[29]
1	10	Barley, wheat, maize, oat, rice	[27]
500	<10	Cereal	[28]

One of the most commonly used technologies so far is the immunochromatographic rapid assay strip test (GIPSA (http://gipsa.usda.gov/GIPSA)). Many tests have been developed for the detection of aflatoxin M1 in milk [30] fumonisins B1 and B2 in maize [31] and aflatoxin B1 in pig feed [32]. One strip-based test kit for OTA gives good performance for wheat and barley and is commercially available according to the GIPSA guidelines. This kit involves a quantitative lateral flow immunoassay with a range of sensitivity of 0 to 150 ppb and a limit of detection of 1 ppb that requires a specific reader: the ROSA^®^ Ochratoxin Quantitative kit is sold with a ROSA-M reader (http://www.charm.com). 

## 4. Flow-Through Tests

**Figure 2:**
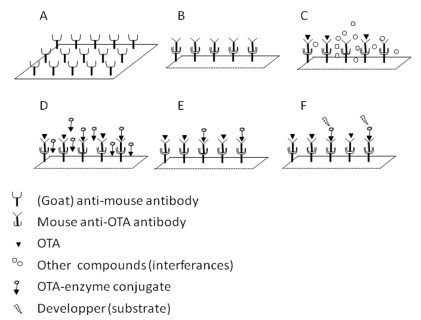
Principle of competitive immunoassay with conjugated toxin, modified from [24]. (**A**) The membrane is coated with the first antibody (usually a goat anti-mouse); (**B**) The second antibody (usually a monoclonal anti-toxin) is then fixed; (**C**) The membrane is then placed in contact with the sample. If the sample contains the specific toxin, the toxin links to the specific antibody; (**D**) A detection element conjugated with the toxin (usually HRP) is then added to the membrane. The amount of conjugated toxin that can be fixed is inversely correlated with the amounts of toxin present in the sample; (**E**) The non-fixed conjugated toxin is rinsed away before adding a developing product (**F**).

Flow-through membrane based immunoassays are comparable with lateral-flow test strips in rapidity and ease of use. However, they are qualitative or semi-quantitative tests, and interpretation of results may be difficult when the sample concentration is close to the cut-off level [33]. These types of devices have been developed for competitive immunoassay detection of various mycotoxins. They are based on modified ELISA tests (Figure 2). 

### Flow-Through: How Does It Work?

**Figure 3:**
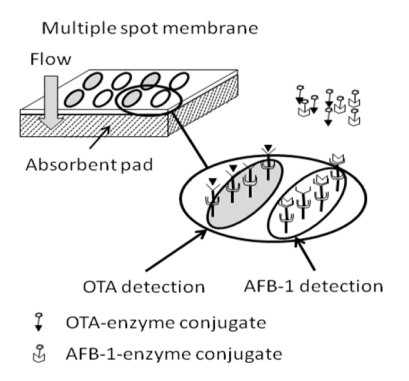
The principle of the simultaneous immunoassay method for aflatoxin B1 (AFB1) and OTA in a flow-through assay. Two types of reagent spots are prepared, one containing the conjugates for AFB1, the other for OTA. The detection process is the same for both samples.

The test principle involves a flow of fluid containing the mycotoxin, through a filter paper (Figure 3). A second layer, or submembrane, inhibits the immediate backflow of fluids, which could corrupt the result. The mycotoxin is captured on the surface of the membrane by a primary antibody and then visualized by the addition of mycotoxin HRP (Horseradish Peroxidase) conjugate. On the membrane, the dot color intensity level is visually compared with a negative control. The most intense color is exhibited by the control because there is an inverse relationship between toxin concentration and color development.

An advantage of this type of device is that it is a very rapid procedure, with results available within 3–5 minutes. A flow-through membrane-based enzyme immunoassay for the rapid detection of OTA in wheat has been developed [35]. The possibility of using this device for the detection of one or multiple mycotoxins including OTA has been explored in samples as diverse as green coffee [36], wheat [35], chili [34], and wine [37]. With the elimination of matrix interferences, this non-instrumental spotting method is sensitive and can quickly estimate the level of contamination of various samples. As shown in Table 3, the LODs obtained are compatible with the regulatory limit established by the European Commission. The limit of this method seems to be the instability of the developed color [38]. In addition, as enzyme activity is temperature and pH dependent, sample preparation, sample volume and clean-up layer processing are crucial steps which can interfere with the quality of the color [24]. Moreover, this method may require densitometric analysis.

**Table 3:** Visual detection limit of different flow-through tests developed for ochratoxin A.

Visual Detection Limit (ng/mL)	Reaction Time (min) *	Sample	References
2	20	Wine	[24]
4	ND	Roasted coffee	[39]
10	ND	Chili	[34]
1	8	Wine, coffee	[37]
2	8	Coffee	[37]

* ND: not determined.

## 5. Clean-Up Tandem Immune Assay Column

There are two main types of clean-up columns, immunoaffinity columns (IAC) and solid phase extraction (SPE) columns. 

IAC represent one of the major cleaning techniques for OTA analysis and that of other mycotoxins [39]. With IAC clean-up, the mycotoxin can be concentrated in the column, thereby increasing the fluorometric assay sensitivity and decreasing its limit of detection. In 2001, Pascale and Visconti used this method for the detection of OTA in urine with a limit of 0.05 ng/mL [33]. Other examples of the potency of this fluorimetric assay have been demonstrated for the detection of OTA in maize [40], in wheat, rice barley raisins, or red wine [41]. Nevertheless, some precaution should be taken. It has been demonstrated by some authors that underestimation could arise when OTA is first extracted in alkaline conditions [42,43,44]. 

As an alternative to IAC application, several variants of SPE have been developed. The use of SPE columns for purification is rapid and profitable. Sibanda *et al.* 2002 optimized this method for rapid detection of OTA in roasted coffee. A one-step SPE clean-up column has been developed for rapid clean-up of mycotoxin for use in a fluorometric method [45,46].

Sample preparation is a crucial step in the determination of OTA and should be kept as simple as possible. However, for heavily colored samples, like red wine and coffee, a simple extraction gives interfering fractions making the development of visual tests more difficult [24]. Moreover, all these techniques require sophisticated equipment and trained staff and cannot be used on-site. 

Hence, there is a need for alternative methods using non-instrumental clean-up up tandem immunoassay columns for the visual detection of OTA. Several such procedures have been developed over the last decade.

### Clean-up Tandem Immune Assay Column: How Does It Work?

**Figure 4:**
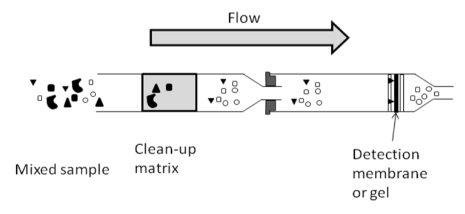
Set-up of the flow-through column connected with a clean-up column.

All rapid systems for the clean-up tandem immunoassay combine sample clean-up and analyte detection based on the direct competitive immunoassay principle (Figure 4). For this, the analyte binds to the antibody immobilized on the gel or membrane in the flow-through column; the added enzyme labeled conjugate can only bind to specific antibodies if they are not occupied by the analyte. Consequently, the amount of bound conjugate and, therefore, the intensity of the developed color are inversely proportional to analyte concentration. When OTA is present, no color develops. There are several types of clean-up columns: the reactive device being in the top or bottom of the column, the filling being made of Bio-Sil NH2 or SAX clean-up layer (retaining impurities and color). However, all clean-up immunoassays use OTA-HRP conjugated for the colorimetric detection. For example, to optimize the detection of OTA in red wine, a SAX clean-up layer was used and the detection layer was washed with PBS to remove residual matrix color in order to be able to distinguish between a blank control and a sample spiked at 2 ng/mL [24]. The visual detection limits of different clean-up tandem immunoassay columns tests developed for OTA can vary from 2 to 10 ng/mL depending on food matrices (Table 4).

**Table 4:** Visual detection limit of different clean-up tandem immunoassay columns tests developed for ochratoxin A.

Visual Detection Limit (ng/mL)	Reaction Time (min) *	Sample	References
2	20	Wine	[24]
6	ND	Roasted coffee	[37]
10	40	Chili red pepper, pili-pili, paprika, ginger	[47]
6	15	Roasted coffee	[48]
2	30	Cocoa powder	[49]
10	10	Highly colored herbs, spices	[47]
0.2	30	Beer	[50]

* ND: not determined.

## 6. Conclusions

Different requirements need to be fulfilled in order to obtain a usable colorimetric test for rapid mycotoxin screening. In this review, three rapid colorimetric devices have been compared: the lateral flow strip test, the flow-through test and the clean-up tandem immunoassay. Membranes offer several advantages: antibodies are covalently bound to the pad, and a simple solvent extraction is used for faintly colored matrices, followed by a filtration step. However, for intensely colored food matrices such as wine, coffee, cocoa, spices, this simple extraction is not sufficient. The clean-up tandem immunoassay, a new system that combines clean-up and detection in a single test device, offers the possibility to develop rapid tests for complex matrices. Despite a strong demand, very few rapid test kits are commercially available. GIPSA has validated only two rapid test kits for OTA detection in non-colored food matrices such as wheat and barley: ROSA^®^ Ochratoxin Quantitative kit, a lateral flow strip (Charm Sciences Inc.) and the OchraTest^®^, a clean-up affinity column (Vicam). Other systems published in scientific journals have probably encountered problems of profitability, usability or reliability. All the preparation steps with antibodies, for example for the detection layer, are sensitive issues and can compromise the robustness of the test [38]. The ideal ochratoxin A test should be a very sensitive portable test, able to give an accurate and rapid answer, usable by a non-scientific technician, reliable, inexpensive, able to overcome the problem of complex/colored matrix, and one that gives a simple visual signal; but such a test has yet to be developed.
